# Effectiveness and Safety of Hypofractionated Radiotherapy in Patients With Ductal Carcinoma In Situ (DCIS)

**DOI:** 10.1155/tbj/9456822

**Published:** 2026-06-08

**Authors:** Reza Samiee, Mohammadamin Kharaghani, Amirhossein Shahsavand, Shayan Forghani, Mehrdad Mozafar, Negin Safari Dehnavi, Zahra Ghanbari, Luca Nicosia, Fatemeh Jafari

**Affiliations:** ^1^ Department of Medicine, School of Medicine, Tehran University of Medical Sciences, Tehran, Iran, tums.ac.ir; ^2^ Advanced Radiation Oncology Department, IRCCS Sacro Cuore Don Calabria Hospital, Negrar Di Valpolicella, Verona, Italy, sacrocuore.it; ^3^ Department of Radiation Oncology, Cancer Institute, Imam Khomeini Hospital Complex, Tehran University of Medical Sciences, Tehran, Iran, tums.ac.ir

**Keywords:** breast-conserving surgery, ductal carcinoma in situ, hypofractionated radiotherapy, meta-analysis, whole-breast irradiation

## Abstract

**Objective:**

To synthesize the available evidence on the oncologic outcomes, toxicity, and cosmesis of hypofractionated whole‐breast irradiation (HF‐WBI) in patients with ductal carcinoma in situ (DCIS) following breast‐conserving surgery (BCS).

**Methods:**

A systematic review and meta‐analysis was conducted according to PRISMA guidelines. PubMed, Embase, and Web of Science were searched from inception until July 12, 2025. We included studies of DCIS patients treated with BCS followed by HF‐WBI (fraction size > 2.0 Gy). Both comparative studies (against conventional fractionation [CF]) and single‐arm studies reporting outcomes for HF‐WBI alone were included. Pooled incidence rates for outcomes from single‐arm studies and pooled hazard ratios (HRs) or odds ratios (ORs) from the limited comparative studies were calculated using random‐effects models.

**Results:**

Nineteen studies were included. Local control and overall survival were excellent and equivalent between HF‐WBI and CF‐WBI. The pooled HR for local recurrence comparing ultrahypofractionation to CF was 0.89 (95% CI 0.64–1.24). HF‐WBI significantly reduced the odds of acute dermatitis (OR 0.22, 95% CI 0.13–0.35). Late toxicities were infrequent (e.g., telangiectasia: 2%, 95% CI 0%–5%). Good/excellent cosmesis was reported in 90% (95% CI 84%–94%) of patients.

**Conclusion:**

HF‐WBI is a safe and effective treatment for DCIS, achieving oncologic outcomes equivalent to CF while offering improved tolerability, reduced acute skin toxicity, and excellent cosmetic results. These benefits, combined with increased convenience and potential cost savings, support the integration of HF‐WBI into standard practice for DCIS.

## 1. Introduction

Ductal carcinoma in situ (DCIS) is a noninvasive stage of breast cancer characterized by malignant epithelial cell proliferation confined within the mammary ducts [1]. Its incidence has risen notably with the expansion of mammographic screening programs, currently accounting for more than 30% of screen‐detected breast malignancies [2]. While DCIS lacks the capacity to metastasize, it is recognized as a precursor to invasive breast cancer (IBC), possessing a potential for progression if left untreated [1]. Consequently, the standard of care generally involves active treatment, most commonly breast‐conserving surgery (BCS) followed by adjuvant whole‐breast irradiation (WBI) [3]. Randomized trials have consistently shown that the addition of WBI significantly diminishes the risk of subsequent ipsilateral breast events (IBEs), encompassing both DCIS recurrence and the development of new IBC [4]. Traditionally, WBI was administered using conventionally fractionated (CF) radiotherapy, typically 50 Gy in 25 daily fractions over five weeks, sometimes supplemented by a sequential tumor bed boost [5]. However, the prolonged duration of conventional radiotherapy can impose logistical and psychosocial burdens on patients and may limit treatment accessibility [6].

For early‐stage IBC, moderately hypofractionated WBI (HF‐WBI), using condensed schedules (e.g., 40–42.5 Gy in 15–16 fractions) is an established standard, offering oncologic outcomes and toxicity profiles comparable or favorable to conventionally fractionated WBI (CF‐WBI) [7]. Observational data suggested HF‐WBI safety and efficacy for DCIS [8, 9]. The DBCG HYPO randomized controlled trial (RCT), including 246 DCIS patients, showed noninferiority of 40 Gy/15 fractions versus 50 Gy/25 fractions for 9‐year local control and morbidity [10]. Concurrently, the BIG 3‐07/TROG 07.01 RCT established equivalent 5‐year local control between HF‐WBI (42.5 Gy/16 fractions) and CF‐WBI (50 Gy/25 fractions) in non–low‐risk DCIS [11]. While moderate HF‐WBI has been increasingly adopted for DCIS based on emerging clinical evidence, uncertainties remain regarding its long‐term oncologic outcomes, optimal integration of boost techniques, and the role of newer ultrahypofractionated regimens across diverse clinical settings. These gaps highlight the need for a comprehensive synthesis of the available evidence.

Therefore, we conducted this systematic review and meta‐analysis to evaluate the current evidence on the effectiveness (local control, survival) and safety (toxicity, cosmesis) of HF‐ versus CF‐WBI regimens for DCIS. This analysis aims to consolidate findings across various hypofractionation strategies, including moderate, ultrahypofractionated, and boost/SIB techniques, providing estimates to inform clinical practice and patient decision‐making.

## 2. Methods

### 2.1. Protocol and Registration

This systematic review and meta‐analysis was conducted and reported in line with the Preferred Reporting Items for Systematic Reviews and Meta‐Analyses (PRISMA) guidelines [12]. The study protocol was prospectively registered with the International Prospective Register of Systematic Reviews (PROSPERO) under registration number CRD420251103185.

### 2.2. Eligibility Criteria

We included studies that enrolled patients with a diagnosis of DCIS treated with BCS followed by adjuvant WBI. Interventions of interest were HF‐WBI, defined as regimens with a fraction size > 2.0 Gy. This included moderate hypofractionation (e.g., 40–42.5 Gy in 15–16 fractions) and ultrahypofractionation (e.g., 26–30 Gy in 5 fractions). Comparators were CF‐WBI, typically defined as 50 Gy in 25 fractions. Eligible study designs comprisedRCTs, prospective and retrospective cohort studies, and single‐arm trials. Studies were required to report on at least one of the following outcomes: local recurrence, overall survival (OS), breast cancer–specific survival (BCSS), distant metastasis, regional nodal recurrence, acute toxicity (e.g., dermatitis, pain, edema, and fatigue), late toxicity (e.g., telangiectasia, induration, and fibrosis), and cosmetic outcomes (physician‐ or patient‐reported). We excluded reviews, case reports, conference abstracts without full data, studies with overlapping populations, and studies focusing solely on partial breast irradiation or brachytherapy.

### 2.3. Information Sources and Search Strategy

A systematic literature search was performed across three electronic databases: PubMed, Embase, and Web of Science, from inception until July 12, 2025. The search strategy combined Medical Subject Headings (MeSH) terms and keywords related to “ductal carcinoma in situ,” “DCIS,” “hypofractionated radiotherapy,” “ultrahypofractionation,” “short‐course,” and “whole breast irradiation.” No language restrictions were applied. The complete search strategy for all databases is provided as an example in Table S1. Reference lists of all included studies and relevant review articles were manually screened to identify any additional eligible publications.

### 2.4. Study Selection and Data Extraction

Search results from the databases were combined and imported into EndNote software (Version 22.3) for deduplication. The subsequent study selection process was carried out independently by two reviewers (R.S. and M.K.) in two phases: (1) screening of titles and abstracts and (2) assessment of full‐text articles against the eligibility criteria. Any disagreements were resolved through discussion or by adjudication from a third senior reviewer (F.J.).

Data were extracted independently by two reviewers (S.F. and A.S.) using a prepiloted, standardized data extraction form in Microsoft Excel. The extracted data included study characteristics (first author, publication year, country, study design, and sample size), patient and tumor characteristics (mean/median age, DCIS grade, margin status, and use of endocrine therapy), intervention details (radiotherapy regimen (total dose, number of fractions, and dose per fraction), use of a tumor bed boost (sequential or simultaneous integrated boost [SIB]), radiotherapy technique (3D‐CRT, IMRT, and VMAT)), and outcome data (for oncologic outcomes, local recurrence, OS, BCSS, distant metastasis, and regional recurrence). We extracted event counts, incidence rates, and hazard ratios (HRs) with 95% confidence intervals (CIs) where available. For toxicity and cosmetic outcomes, we extracted event counts and proportions for each grade. The follow‐up duration for each outcome was also recorded.

### 2.5. Quality Assessment

Two independent reviewers (R.S. and A.H.) evaluated the methodological quality of the included studies using the Joanna Briggs Institute (JBI) critical appraisal tools [13]. Depending on the study design, the following checklists were applied: the Checklist for Cohort Studies for observational research, the Risk of Bias Tool for RCTs for experimental studies, and the Checklist for Case Series for descriptive clinical evidence. Any discrepancies in scoring were resolved through consensus discussions among all authors.

### 2.6. Data Synthesis and Statistical Analysis

All statistical analyses were performed using R software (Version 4.4.2) with the “meta” and “metafor” packages. For single‐arm studies, pooled incidence rates for toxicity and oncologic outcomes were calculated using a random‐effects model with the DerSimonian and Laird method. Proportions were stabilized using the Freeman–Tukey double arcsine transformation. Heterogeneity was quantified using the *I* ^2^ statistic, with values of 25%, 50%, and 75% representing low, moderate, and high heterogeneity, respectively.

**FIGURE 1:**
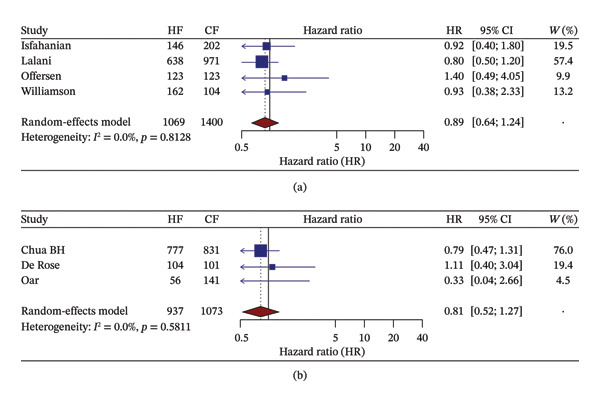
Forest plots of pooled hazard ratios comparing hypofractionated versus conventional fractionation radiotherapy for (a) local recurrence and (b) overall survival in DCIS patients.

For comparative analyses (HF‐WBI vs. CF‐WBI), pooled HRs for time‐to‐event outcomes (local recurrence and OS) and pooled odds ratios (ORs) for dichotomous outcomes (acute dermatitis, pain, and cosmesis) were calculated using a random‐effects model (inverse variance method for HRs and Mantel–Haenszel method for ORs). Only studies that reported HRs, or had enough data to calculate them, were included in the quantitative analysis of time‐to‐event outcomes; therefore, the meta‐analyses for local recurrence and OS shown in Figure 1 include only these studies. A pooled effect estimate with a 95% CI not crossing 1.0 was considered statistically significant.

Prespecified subgroup analyses and univariate meta‐regression were conducted to explore sources of heterogeneity for key outcomes, including acute dermatitis and telangiectasia. Covariates investigated included study design (RCT vs. retrospective), radiotherapy technique (3D‐CRT vs. VMAT), number of fractions, biological effective dose (BED), equivalent dose in 2 Gy fractions (EQD2), and the proportion of patients receiving a boost or hormone therapy. For continuous variables such as BED and EQD2, values were first calculated for each whole‐breast radiotherapy schedule using the linear‐quadratic model assuming an *α*/*β* ratio of 4 Gy, and studies were subsequently categorized into “high” and “low” groups based on the median values across the included cohorts to facilitate subgroup analyses and meta‐regression.

Sensitivity analyses were performed by sequentially excluding each study to assess the robustness of the pooled estimates. Publication bias was planned to be assessed visually using funnel plots and statistically using Egger’s test for outcomes including 10 or more studies. All *p* values were two‐sided, and statistical significance was set at *p* < 0.05.

## 3. Results

### 3.1. Study Selection and Characteristics

The systematic literature search identified 2057 records from the electronic databases. After duplicate removal and a two‐stage screening process of titles, abstracts, and full texts against the eligibility criteria, 19 studies were included in the quantitative synthesis. The study selection process is detailed in the PRISMA flow diagram (Figure S1). The included studies comprised RCTs, prospective and retrospective cohorts, and single‐arm trials, with their key characteristics summarized in Table 1. The methodological quality assessment of the included studies, performed using JBI critical appraisal tools, is presented in Figure S2.

**TABLE 1:** Characteristics of included studies.

First author (year)	Country	Study design	Arm/fractionation schedule	Sample size (n)	Median/mean age (years)	Boost (%)	RT technique	Median follow‐up (years)
Chua et al. [11] (2022)	Multinational	RCT	CF‐WBI (50 Gy/25fx)	831	—	—	3D‐CRT	6.6
			HF‐WBI (42.5 Gy/16 fx)	777	—	—	3D‐CRT	

Guenzi et al. [14] (2013)	Italy	Retrospective cohort	CF‐WBI (46 Gy/20 fx)	41	—	82.9%	3D‐CRT	2.5
			HF‐WBI (39 Gy/13 fx)	72	—	90.3%	3D‐CRT	

Karasawa et al. [15] (2012)	Japan	Prospective cohort	CF‐WBI (50 Gy/25 fx)	381	Median: 53 (range 22–88)	40.9%	3D‐CRT	2.3
			HF‐WBI (43.2 Gy/16 fx)	717	Median: 54 (range 29–85)	32.9%	3D‐CRT	

Isfahanian et al. [16] (2016)	Canada	Retrospective cohort	CF‐WBI (50 Gy/25 fx)	202	Median: 57 (range 36–84)	23%	—	5.6
			HF‐WBI (42.6 Gy (75%) or 45 Gy (25%)/16 fx (75%) or 18 fx (25%))	146	Median: 61 (range 32–86)	31%	—	5.3

Lalani et al. [17]. (2014)	Canada	Retrospective cohort	CF‐WBI (50 Gy/25 fx)	971	Median: 55 (IQR 49–64)	14.7%	—	9.1
			HF‐WBI (40–44 Gy/16 fx)	638	Median: 57 (IQR 50–66)	54.2%	—	9.4

Oar et al. [18] (2016)	Australia	Retrospective cohort	CF‐WBI (45 Gy (17.0%), 46 Gy (6.4%), or 50 Gy (76.6%)/25 fx (17.0%), 23 fx (6.4%), or 25 fx (76.6%))	141	Median: 57.2	83%	3D‐CRT	4.4
			HF‐WBI (42.2–42.6 Gy/16 fx)	56	Median: 57	48%	3D‐CRT	4.3

Williamson et al. [19] (2010)	Canada	Retrospective cohort	CF‐WBI (50 Gy/25 fx)	104	Mean: 56.5 ± 9.6	0%	3D‐CRT	3.8
			HF‐WBI (42.4 Gy (73%) or 40 Gy (27%)/16 fx)	162	Mean: 58.4 ± 9.3	27%	3D‐CRT	

De Rose et al. [8] (2020)	Italy	Retrospective cohort	CF‐WBI (50 Gy/25 fx)	101	Median: 58 (range 30–87)	0%	—	12.6
			HF‐WBI (40.5 Gy/15 fx)	104	Median: 57 (range 30–87)	0%	—	1.8

Offersen et al. [10] (2020)	Denmark, Germany, Norway	RCT	CF‐WBI (50 Gy/25 fx)	123	Median: 59 (range 42–83)	81%	3D‐CRT	7.3
			HF‐WBI (40 Gy/15 fx)	123	Median: 59 (range 41–82)	82%	3D‐CRT	

Pinnarò et al. [20] (2017)	Italy	Single‐arm trial	HF‐WBI (34 Gy/10 fx)	251	Mean: 61.1 ± 12.2	100%	3D‐CRT	5.4

Unterkirhere et al. [21] (2023)	Switzerland	Prospective cohort	HF‐WBI (40.05 Gy/15 fx)	33	—	100%	—	2.8

De Rose et al. [22] (2018)	Italy	Retrospective cohort	HF‐WBI (40.5 Gy/15 fx)	137	Median: 58 (range 30–87)	0%	VMAT	1.8

Soonthornrak et al. [23] (2022)	Thailand	Single‐arm trial	HF‐WBI (42.5 Gy/16 fx)	33	Median: 56 (range 36–74)	100%	IMRT	0.6

Berlin et al. [24] (2019)	USA	Single‐arm trial	HF‐WBI (40.5 Gy/15 fx)	104	Median: 59 (range 37–85)	100%	—	6.2

Constantine et al. [25] (2008)	USA	Single‐arm trial	HF‐WBI (42 Gy/15 fx)	59	Median: 54 (range 36–78)	0%	3D‐CRT	3.0

Livi et al. [26] (2007)	Italy	Retrospective cohort	HF‐WBI (44 Gy/16 fx)	48	—	—	3D‐CRT	4.3

Cante et al. [27] (2014)	Italy	Prospective cohort	HF‐WBI (45 Gy/20 fx)	103	Median: 62.1	100%	3D‐CRT	4.0

Ciervide et al. [28] (2012)	USA	Single‐arm trial	HF‐WBI with Boost (40.5 Gy/15 fx)	86	—	100%	3D‐CRT/IMRT	3.2
			HF‐WBI without Boost (42 Gy/15 fx)	59	—	0%	3D‐CRT/IMRT	6.0

Hathout et al. [29] (2013)	Canada	Retrospective cohort	HF‐WBI without Boost (42.5 Gy/16 fx)	315	—	0%	3D‐CRT	4.4
			HF‐WBI without Boost (42.5 Gy/16 fx)	125	—	100%	3D‐CRT	

### 3.2. Overall Toxicity and Cosmetic Outcomes

The pooled incidence of acute toxicities from studies of HF‐WBI was generally low. For acute grade ≥ 2 skin dermatitis, the meta‐analysis of 8 studies (*n* = 1362 patients) yielded a pooled incidence of 7% (95% CI 4%–12%), albeit with significant heterogeneity (*I* ^2^ = 84.6%, *p* < 0.001). The incidence of acute grade ≥ 2 edema (3 studies, *n* = 234) was 8% (95% CI 5%–14%), with moderate heterogeneity (*I* ^2^ = 46.0%). Acute grade ≥ 2 pain was reported in 4% of patients (95% CI 1%–17%; 7 studies, *n* = 1259), though with high heterogeneity (*I* ^2^ = 93.4%), while grade ≥ 2 fatigue (2 studies, *n* = 163) was rare at 1% (95% CI 0%–4%) without heterogeneity.

Regarding late toxicities, the pooled incidence of grade ≥ 2 telangiectasia across 6 studies (*n* = 759 patients) was 2% (95% CI 0%–5%), with significant heterogeneity (*I* ^2^ = 67.0%, *p* = 0.010). Other late toxicities were infrequent: any‐grade hyperpigmentation (4 studies, *n* = 267) had a pooled incidence of 6% (95% CI 0%–62%; *I* ^2^ = 95.9%), grade ≥ 2 induration (6 studies, *n* = 541) was 2% (95% CI 1%–4%; *I* ^2^ = 5.6%), and breast shrinkage (2 studies, *n* = 175) was 4% (95% CI 0%–63%; *I* ^2^ = 85.6%). The incidence of symptomatic grade ≥ 2 pneumonitis or lung fibrosis was negligible at 0% (95% CI 0%–1%; 2 studies, *n* = 820).

Cosmetic outcomes from studies were excellent, with a pooled estimate for good/excellent cosmesis of 90% (95% CI 84%–94%; 8 studies, *n* = 848), though heterogeneity was present (*I* ^2^ = 81.3%) (Table 2).

**TABLE 2:** Pooled and subgroup analysis of outcomes between high and low BED versus EQD2 in breast cancer radiotherapy.

Outcome	Metric	Subgroup	Number of studies	Proportion [95% CI]	*I* ^2^ (%)	*p* value (subgroup diff.)
Local recurrence (5‐year)	Pooled	Overall	9	0.06 [0.05; 0.07]	0	—
	BED	High	4	0.07 [0.05; 0.08]	0	0.365
		Low	5	0.05 [0.04; 0.08]	0	
	EQD2	High	4	0.07 [0.05; 0.08]	0	0.365
		Low	5	0.05 [0.04; 0.08]	0	

Local recurrence (3‐year)	Pooled	Overall	12	0.03 [0.02; 0.04]	43.6	—
	BED	High	5	0.01 [0.00; 0.05]	69.1	0.186
		Low	7	0.04 [0.02; 0.05]	0	
	EQD2	High	5	0.01 [0.00; 0.05]	69.1	0.186
		Low	7	0.04 [0.02; 0.05]	0	

Overall survival (3‐year)	Pooled	Overall	5	0.99 [0.97; 1.00]	0	—
	BED	High	2	1.00 [0.94; 1.00]	0	0.933
		Low	3	0.99 [0.95; 1.00]	0	
	EQD2	High	2	1.00 [0.94; 1.00]	0	0.933
		Low	3	0.99 [0.95; 1.00]	0	

Breast cancer–specific mortality (3‐year)	Pooled	Overall	5	0.01 [0.00; 0.01]	0	—
	BED	High	3	0.00 [0.00; 0.01]	0	0.582
		Low	2	0.01 [0.00; 0.06]	0	
	EQD2	High	3	0.00 [0.00; 0.01]	0	0.582
		Low	2	0.01 [0.00; 0.06]	0	

Distant metastasis (3‐year)	Pooled	Overall	11	0.01 [0.01; 0.02]	0	—
	BED	High	5	0.01 [0.01; 0.02]	0	0.533
		Low	6	0.01 [0.01; 0.03]	0	
	EQD2	High	5	0.01 [0.01; 0.02]	0	0.533
		Low	6	0.01 [0.01; 0.03]	0	

Distant metastasis (5‐year)	Pooled	Overall	7	0.02 [0.01; 0.03]	8.8	—
	BED	High	4	0.01 [0.00; 0.02]	0	0.074
		Low	3	0.03 [0.01; 0.05]	0	
	EQD2	High	4	0.01 [0.00; 0.02]	0	0.074
		Low	3	0.03 [0.01; 0.05]	0	

Regional nodal recurrence (3‐year)	Pooled	Overall	10	0.01 [0.00; 0.03]	70.1	—
	BED	High	6	0.00 [0.00; 0.01]	0	**< 0.001**
		Low	4	0.06 [0.04; 0.09]	26.1	
	EQD2	High	6	0.00 [0.00; 0.01]	0	**< 0.001**
		Low	4	0.06 [0.04; 0.09]	26.1	

Regional nodal recurrence (5‐year)	Pooled	Overall	5	0.00 [0.00; 0.02]	0	—
	BED	High	4	0.00 [0.00; 0.02]	0	0.909
		Low	1	0.00 [0.00; 0.04]	—	
	EQD2	High	4	0.00 [0.00; 0.02]	0	0.909
		Low	1	0.00 [0.00; 0.04]	—	

Cosmetic outcome	Pooled	Overall	8	0.90 [0.84; 0.94]	81.3	—
	BED	High	4	0.88 [0.77; 0.95]	71.8	0.492
		Low	4	0.92 [0.84; 0.96]	86.1	
	EQD2	High	4	0.88 [0.77; 0.95]	71.8	0.492
		Low	4	0.92 [0.84; 0.96]	86.1	

Dermatitis	Pooled	Overall	8	0.07 [0.04; 0.12]	84.6	—
	BED	High	4	0.06 [0.03; 0.11]	73.1	0.637
		Low	4	0.08 [0.03; 0.20]	76.4	
	EQD2	High	4	0.06 [0.03; 0.11]	73.1	0.637
		Low	4	0.08 [0.03; 0.20]	76.4	

Pain	Pooled	Overall	7	0.04 [0.01; 0.17]	93.4	—
	BED	High	3	0.01 [0.00; 0.02]	0	**0.004**
		Low	4	0.12 [0.02; 0.41]	86.7	
	EQD2	High	3	0.01 [0.00; 0.02]	0	**0.004**
		Low	4	0.12 [0.02; 0.41]	86.7	

Telangiectasia	Pooled	Overall	6	0.02 [0.00; 0.05]	67.0	—
	BED	High	2	0.02 [0.00; 0.05]	0	0.850
		Low	4	0.01 [0.00; 0.08]	73.2	
	EQD2	High	2	0.02 [0.00; 0.05]	0	0.850
		Low	4	0.01 [0.00; 0.08]	73.2	

Hyperpigmentation	Pooled	Overall	4	0.06 [0.00; 0.62]	95.9	—
	BED	High	3	0.12 [0.00; 0.87]	96.7	0.224
		Low	1	0.00 [0.00; 0.05]	—	
	EQD2	High	3	0.12 [0.00; 0.87]	96.7	0.224
		Low	1	0.00 [0.00; 0.05]	—	

Induration	Pooled	Overall	6	0.02 [0.01; 0.04]	5.6	—
	BED	High	3	0.02 [0.01; 0.04]	5.6	0.250
		Low	3	0.03 [0.00; 0.10]	28.6	
	EQD2	High	3	0.02 [0.01; 0.04]	5.6	0.250
		Low	3	0.03 [0.00; 0.10]	28.6	

*Note:* BED and EQD2 values correspond to the whole‐breast irradiation component of each regimen and were calculated using the linear‐quadratic model assuming an α/β ratio of 4 Gy for breast tissue. Regimens were categorized as high or low BED/EQD2 according to the median values across the included studies. Values in bold are statistically significant (*p* < 0.05).

### 3.3. Oncologic Outcomes

Oncologic outcomes for patients receiving HF‐WBI were highly favorable. The 3‐year OS rate was 99% (95% CI 97%–100%; 5 studies, *n* = 386), and the 5‐year OS was 98% (95% CI 96%–99%; 6 studies, *n* = 603), both without heterogeneity. Breast cancer–specific mortality was very low at both 3 years (1%, 95% CI 0%–1%; 5 studies, *n* = 954) and 5 years (1%, 95% CI 0%–4%; 3 studies, *n* = 201). Local control rates were high, with 3‐year and 5‐year local recurrence rates of 3% (95% CI 2%–4%; 12 studies, *n* = 1691) and 6% (95% CI 5%–7%; 9 studies, *n* = 1613), respectively. The 3‐year and 5‐year distant metastasis rates were 1% (95% CI 1%–2%; 11 studies, *n* = 1876) and 2% (95% CI 1%–3%; 7 studies, *n* = 939), respectively. Regional nodal recurrence was 1% at 3 years (95% CI 0%–3%; 10 studies, *n* = 1716) and 0% at 5 years (95% CI 0%–2%; 5 studies, *n* = 633) (Table 2). Sensitivity analyses, including leave‐one‐out procedures and restriction to studies reporting time‐to‐event estimates, did not materially change the pooled 3‐ and 5‐year local recurrence rates, supporting the robustness of these findings.

### 3.4. Comparative Outcomes: Hypofractionation vs. CF

#### 3.4.1. Efficacy

A meta‐analysis of four studies (*n* = 2469 patients) comparing HF to CF for local recurrence demonstrated no significant difference between the two approaches. The pooled HR was 0.89 (95% CI 0.64–1.24), indicating no increased risk of local recurrence with hypofractionation (Figure 1a). No heterogeneity was observed among these studies (*I* ^2^ = 0.0%, *p* = 0.81). Similarly, a meta‐analysis of three studies (*n* = 2010 patients) showed no significant difference in OS between HF and CF regimens. The pooled HR was 0.81 (95% CI 0.52–1.27) (Figure 1b), with no heterogeneity (*I* ^2^ = 0.0%, *p* = 0.58).

#### 3.4.2. Toxicity and Cosmesis

Comparative analysis revealed a significant reduction in the odds of acute grade ≥ 2 dermatitis with hypofractionation. The pooled OR from two studies (*n* = 1211 patients) was 0.22 (95% CI 0.13–0.35), favoring the HF group with no heterogeneity (*I* ^2^ = 0.0%). There was no significant difference in the odds of acute grade ≥ 2 pain between the HF and CF groups. The pooled OR from two studies (*n* = 1211 patients) was 1.50 (95% CI 0.39–5.82), with no heterogeneity (*I* ^2^ = 0.0%). The comparative analysis for good/excellent cosmetic outcomes showed no significant difference between groups (OR 1.21, 95% CI 0.01–173.80). However, this result was derived from only two studies (*n* = 310) and was accompanied by considerable heterogeneity (*I* ^2^ = 95.1%).

#### 3.4.3. Subgroup and Meta‐Regression Analyses

Subgroup analyses were conducted to explore sources of heterogeneity for key outcomes (Tables S2, S3, S4, and S5). For acute dermatitis from single‐arm studies, a significantly higher incidence was observed in retrospective cohorts (15%, 95% CI 11%–19%) compared to clinical trials (5%, 95% CI 3%–10%), and with VMAT (15%, 95% CI 12%–20%) compared to 3D‐CRT (5%, 95% CI 2%–11%) (Table S3).

For telangiectasia, a significant subgroup difference was found based on the number of fractions (*p* = 0.0019), with the highest incidence in the 10‐fraction regimen (7%, 95% CI 5%–11%; Table S5). Meta‐regression for telangiectasia identified several significant predictors: a higher proportion of patients receiving hormone therapy (*p* = 0.008), the use of a boost (*p* = 0.042), and a higher proportion of Grade 3 tumors (*p* = 0.049) were associated with increased incidence. Larger sample size was associated with a lower reported incidence (*p* < 0.001; Table S6).

Furthermore, meta‐regression for 3‐year regional nodal recurrence showed that higher BED (*p* < 0.001) and EQD2 (*p* < 0.001) scores were significantly associated with a lower recurrence incidence (Table S6). This finding was corroborated by the subgroup analysis in Table 2, which demonstrated a significantly lower 3‐year regional nodal recurrence rate in the high BED/EQD2 subgroup (0%, 95% CI 0%–1%) compared to the low BED/EQD2 subgroup (6%, 95% CI 4%–9%; *p* < 0.001). For acute pain, both a higher BED (*p* = 0.025) and a higher proportion of patients receiving hormone therapy (*p* < 0.001) were associated with a lower reported incidence (Table S6).

## 4. Discussion

Our meta‐analysis shows that HF‐WBI achieves outcomes equivalent to CF‐WBI for DCIS while improving treatment tolerability. This finding is particularly robust as it is strongly supported and extended by evidence from RCTs included in our synthesis. Specifically, the BIG 3‐07/TROG 07.01 trial and the DBCG HYPO trial were pivotal in establishing the noninferiority of moderate HF‐WBI for DCIS, showing comparable long‐term local control and acceptable toxicity profiles [10, 11]. Across nearly 15,000 patients, local control and OS were excellent and statistically similar between regimens. HF‐WBI significantly reduced the risk of acute skin dermatitis and showed comparably low rates of other acute toxicities, including pain, edema, and fatigue. Late toxicities such as telangiectasia and fibrosis were uncommon, and cosmetic results were highly favorable, with good to excellent cosmesis in about 90% of patients. Overall, HF‐WBI offers a safe, effective, and more convenient alternative to conventional schedules, minimizing treatment burden without compromising efficacy.

In early‐stage IBC, several major trials have shown that shorter radiotherapy schedules work just as well as traditional ones. The UK START A/B trials proved that giving 40 Gy in 15 fractions provided the same 10‐year local control as 50 Gy in 25 fractions, with fewer late side effects [30]. The Canadian OCOG trial also confirmed that 42.5 Gy in 16 fractions was as effective as 50 Gy in 25 fractions, with good cosmetic results [31]. The FAST trial tested once‐weekly treatment and found that 28.5 Gy in 5 fractions was safe and effective [7]. The large FAST‐Forward trial then demonstrated that 26 Gy in 5 fractions over one week was noninferior to 40 Gy in 15 fractions, establishing it as a modern, convenient standard [32].

In line with findings in early‐stage IBC, the efficacy and benefits of hypofractionation have also been demonstrated across various other cancer types. For instance, in prostate cancer, studies have shown that shorter regimens can achieve comparable disease control and toxicity profiles to CF [33, 34]. Similarly, in rectal cancer and certain glioblastoma patient populations, hypofractionated schedules have offered equivalent outcomes while improving patient convenience and treatment tolerability [35, 36].

While the overall evidence strongly supports the safety and efficacy of hypofractionation, not all regimens are equivalent, and some hypofractionated schedules have shown higher late toxicity compared with conventional treatment. In the UK FAST trial, the once‐weekly regimen of 30 Gy in 5 fractions resulted in significantly greater late normal‐tissue effects than the conventional 50 Gy in 25 fractions, whereas 28.5 Gy in 5 fractions produced acceptable outcomes [7]. This finding highlights that even small increases in dose per fraction can meaningfully affect long‐term tissue tolerance. Similarly, in the FAST‐Forward trial, the 27 Gy in 5 fractions arm was associated with more moderate or marked breast induration compared with 40 Gy in 15 fractions, while 26 Gy in 5 fractions achieved comparable results [32].

Similar cautionary lessons have emerged from other cancer types; for example, some prostate cancer trials found certain hypofractionated regimens resulted in higher rates of late genitourinary and gastrointestinal toxicity compared to conventional approaches [37, 38]. This underscores the necessity of careful regimen selection, patient stratification, and long‐term follow‐up to ensure both safety and disease control are preserved.

Taken together, these findings emphasize that the benefits of hypofractionation are context‐dependent. When designed and delivered appropriately, shorter schedules maintain efficacy and improve convenience, but excessive dose per fraction or use in unsuitable clinical settings can increase toxicity. Thus, careful regimen selection, patient stratification, and long‐term follow‐up remain essential to ensure that treatment de‐escalation preserves both safety and disease control.

Radiobiologically, breast tumors behave as relatively low *α*/*β* tissues, with pooled estimates around ∼3–4 Gy, which supports the use of larger fractions without compromising tumor control; this is the same principle that underpins the success of hypofractionation in early‐stage invasive disease. A lower *α*/*β* implies comparable or better biologically effective dose for tumor control at shorter schedules while keeping late‐reacting normal tissues within tolerance [39].

Clinically, shortening overall treatment time (OTT) confers practical and meaningful benefits for patients. Real‐world data show fewer unplanned interruptions and better schedule adherence with hypofractionation: In a breast adjuvant cohort, 13.6% of patients on HF missed ≥ 1 fraction versus 26.7% on CF; CF was the only independent predictor of noncompliance (OR 2.3, 95% CI 1.3–4.2), with longer average interruption duration under CF (3.2 vs. 2.3 days). Because treatment prolongation can adversely affect local control, reducing OTT is not only convenient but potentially protective [40]. Beyond individual adherence, travel burden and distance to treatment facilities are repeatedly linked to delays in radiation initiation and lower utilization; shorter regimens mitigate these barriers, particularly for rural and socioeconomically vulnerable groups [41]. Patient‐centered preference studies further indicate that women place tangible value on shorter radiation courses when trade‐offs in efficacy and side effects are neutral, echoing the everyday priorities we see in clinic (work, caregiving, and transport) [42].

System‐level and financial implications also favor shorter courses. In a large U.S. claims analysis (2008–2013), hypofractionated WBI was associated with lower total 1‐year health expenditures than CF, by $2894 in the guideline‐endorsed cohort and $8587 in the guideline‐permitted cohort, without compromising outcomes [43]. A broader Medicare analysis similarly found ∼33% lower 90‐day radiation‐related spending for breast cancer with shorter regimens [44]. Complementing these payer‐side findings, the FAST‐Forward Health Technology Assessment demonstrated that the 26 Gy in 5 fractions regimen was cost‐saving compared with 40 Gy in 15 fractions from a National Health Service perspective. The analysis estimated mean cost savings of approximately £2002 per patient (95% CI £1245–£2804) and a small gain in quality‐adjusted life years (QALYs) in the base case (0.04 QALYs). Furthermore, by reducing the number of treatment sessions from 15 to 5, the shorter schedule was projected to improve departmental efficiency, decrease treatment‐related resource use, and reduce patient travel and hospital attendance, without compromising clinical outcomes.

This meta‐analysis has several limitations that should be considered when interpreting the findings. First, the included studies were heterogeneous in design, encompassing randomized trials, prospective cohorts, and retrospective analyses, which introduces potential selection and reporting biases. The variability in hypofractionated regimens, dose prescriptions, use of a boost or SIB, and cosmetic assessment tools further contributes to interstudy heterogeneity. Some outcomes, particularly acute toxicity and cosmesis, were based on subjective or nonstandardized grading systems, limiting the precision of pooled estimates. Additionally, certain unexpected associations observed in meta‐regression analyses should be interpreted with caution. These findings often involve small sample sizes or heterogeneous subgroups, limiting their robustness and generalizability. Publication bias cannot be excluded, as studies with negative or neutral findings may be underreported. Importantly, none of the included studies provided follow‐up exceeding 10 years. This absence of long‐term data limits the ability to evaluate late recurrences, long‐term local control, and late normal‐tissue or cosmetic effects, particularly given the prolonged natural history of DCIS. Evidence specific to DCIS remains limited, particularly regarding how hypofractionated schedules interact with other adjuvant treatments and biological tumor characteristics. The influence of concurrent or sequential endocrine therapy on recurrence risk and radiation toxicity has not been well defined, and the potential role of molecular or genomic markers in predicting radiosensitivity or local recurrence warrants further investigation.

Despite these limitations, the study’s strengths include the comprehensive scope of evidence synthesized, the large aggregated sample size exceeding 15,000 patients, and the use of rigorous statistical methods to quantify pooled outcomes across multiple study designs. These features enhance the robustness and generalizability of the findings, providing a reliable overview of the safety, efficacy, and cosmetic results of hypofractionated regimens in DCIS.

Future research should therefore focus on well‐designed, prospective, DCIS‐specific randomized trials comparing moderate and ultrahypofractionated regimens, ideally with standardized reporting of toxicity and cosmetic outcomes. Long‐term follow‐up is essential to verify the sustained efficacy and safety of these schedules. Incorporation of patient‐reported outcome measures (PROMs) will be key to capturing cosmetic satisfaction and quality‐of‐life perspectives. Additionally, translational studies exploring biological predictors of treatment response and recurrence could enable personalized radiotherapy approaches. Finally, health economic and equity analyses are warranted to assess the broader system‐level impact of adopting shorter regimens, particularly in diverse and resource‐limited healthcare settings.

Overall, this meta‐analysis supports HF‐WBI as a safe and effective treatment for DCIS following BCS. HF‐WBI achieves outcomes comparable to CF‐WBI while reducing acute toxicity and maintaining excellent cosmetic results. The consistency of results across different populations and radiotherapy techniques highlights its applicability in clinical practice. However, uncertainties remain regarding ultrahypofractionated regimens, the use of boost or SIB, and long‐term safety beyond 10 years, warranting further DCIS‐specific randomized trials to refine and individualize treatment strategies.

## 5. Conclusion

In conclusion, HF‐WBI achieves equivalent tumor control and survival outcomes compared with conventional schedules, with the added benefits of lower acute toxicity, favorable late effects, and excellent cosmetic outcomes. These advantages, combined with improved treatment convenience and efficiency, support the integration of HF‐WBI into the routine management of DCIS after BCS. Future research should focus on validating ultrahypofractionated regimens, optimizing boost techniques, and extending long‐term follow‐up to confirm durability and safety in this patient population.

## Author Contributions

Conceptualization: Fatemeh Jafari, Luca Nicosia, and Reza Samiee.

Methodology: Reza Samiee, Mohammadamin Kharaghani, and Fatemeh Jafari.

Formal analysis: Reza Samiee, Mohammadamin Kharaghani, and Shayan Forghani.

Investigation: Reza Samiee, Mohammadamin Kharaghani, Amirhossein Shahsavand, Shayan Forghani, Mehrdad Mozafar, Negin Safari Dehnavi, and Zahra Ghanbari.

Data curation: Shayan Forghani and Amirhossein Shahsavand.

Writing–original draft: Reza Samiee, Mohammadamin Kharaghani, and Fatemeh Jafari.

Writing–review and editing: all authors.

Visualization: Reza Samiee and Mohammadamin Kharaghani.

Supervision: Fatemeh Jafari and Luca Nicosia.

Project administration: Fatemeh Jafari.

## Funding

This research did not receive any specific grant from funding agencies in the public, commercial, or not‐for‐profit sectors.

## Ethics Statement

The authors have nothing to report.

## Conflicts of Interest

The authors declare no conflicts of interest.

## Supporting Information

Additional supporting information can be found online in the Supporting Information section.

## Supporting information


**Supporting Information 1** Figure S1. PRISMA 2020 flow diagram for the systematic review.

**Supporting Information 2** Figure S2. Risk of bias assessment of included studies. (a) Randomized controlled trials, (b) single‐arm trials, and (c) cohort studies.

**Supporting Information 3** Table S1. Full search strategy for PubMed, Embase, WoS, and Scopus databases.

**Supporting Information 4** Table S2. Subgroup analysis of toxicities and oncological outcomes by study design.

**Supporting Information 5** Table S3. Subgroup analysis of toxicities and oncological outcomes by radiotherapy technique.

**Supporting Information 6** Table S4. Subgroup analysis of toxicities and oncological outcomes by treatment week.

**Supporting Information 7** Table S5. Subgroup analysis of toxicities and oncological outcomes by fractionation schedule.

**Supporting Information 8** Table S6. Meta‐regression analysis of treatment‐related toxicities and oncological outcomes in breast cancer radiotherapy: association with clinical and dosimetric variables.

## Data Availability

All data have been presented in the manuscript.
