# Supplementary figures and images for: Effectiveness and Safety of Hypofractionated Radiotherapy in Patients With Ductal Carcinoma In Situ (DCIS)
Source: Breast J. 2026 Jun 8;2026:9456822. doi: 10.1155/tbj/9456822 (PMC13244251; doi:10.1155/tbj/9456822)

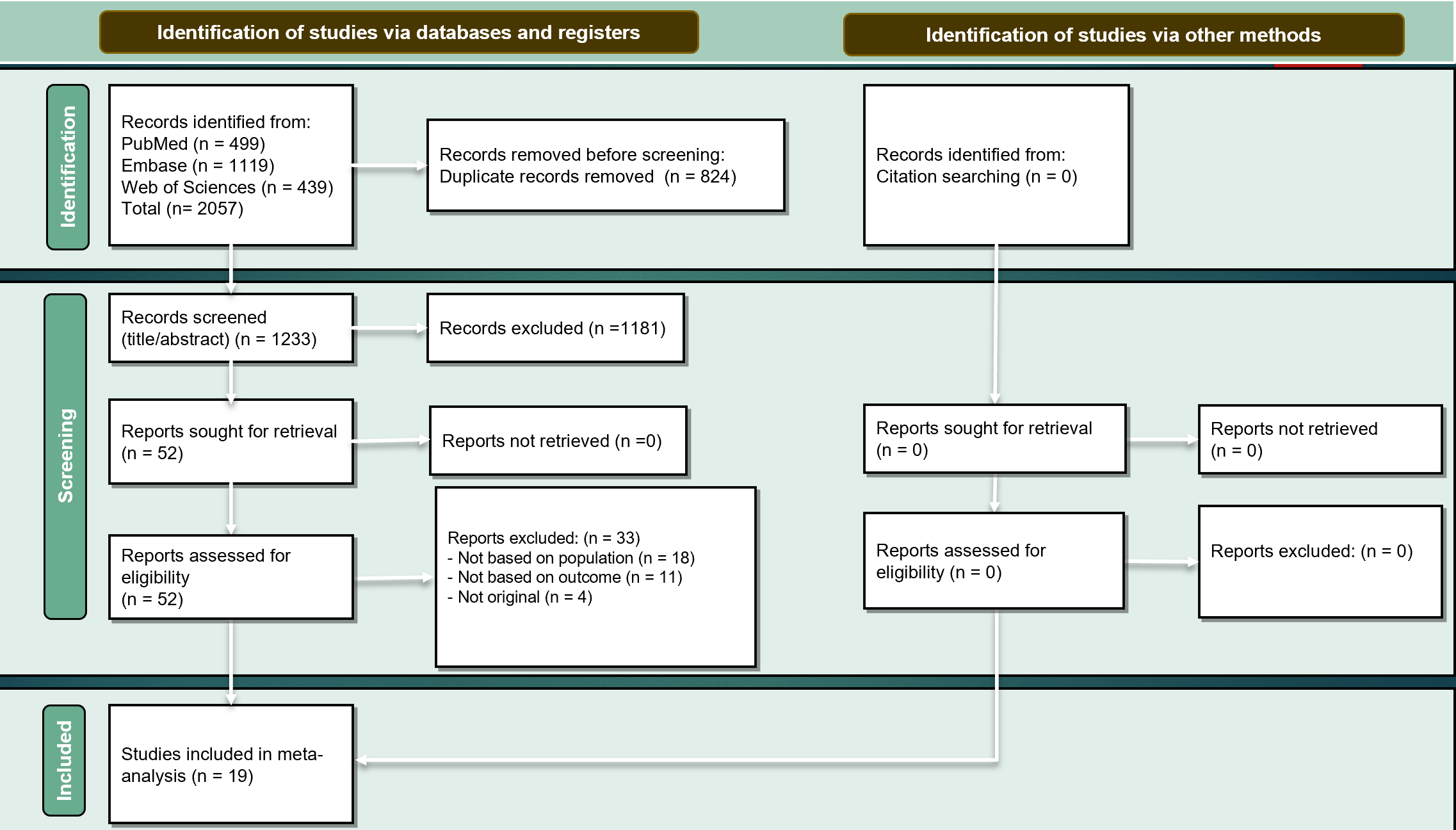

Supplement: Supplementary file 1 — Supporting Information 1 Figure S1. PRISMA 2020 flow diagram for the systematic review. [file TBJ-2026-9456822-s007.png]

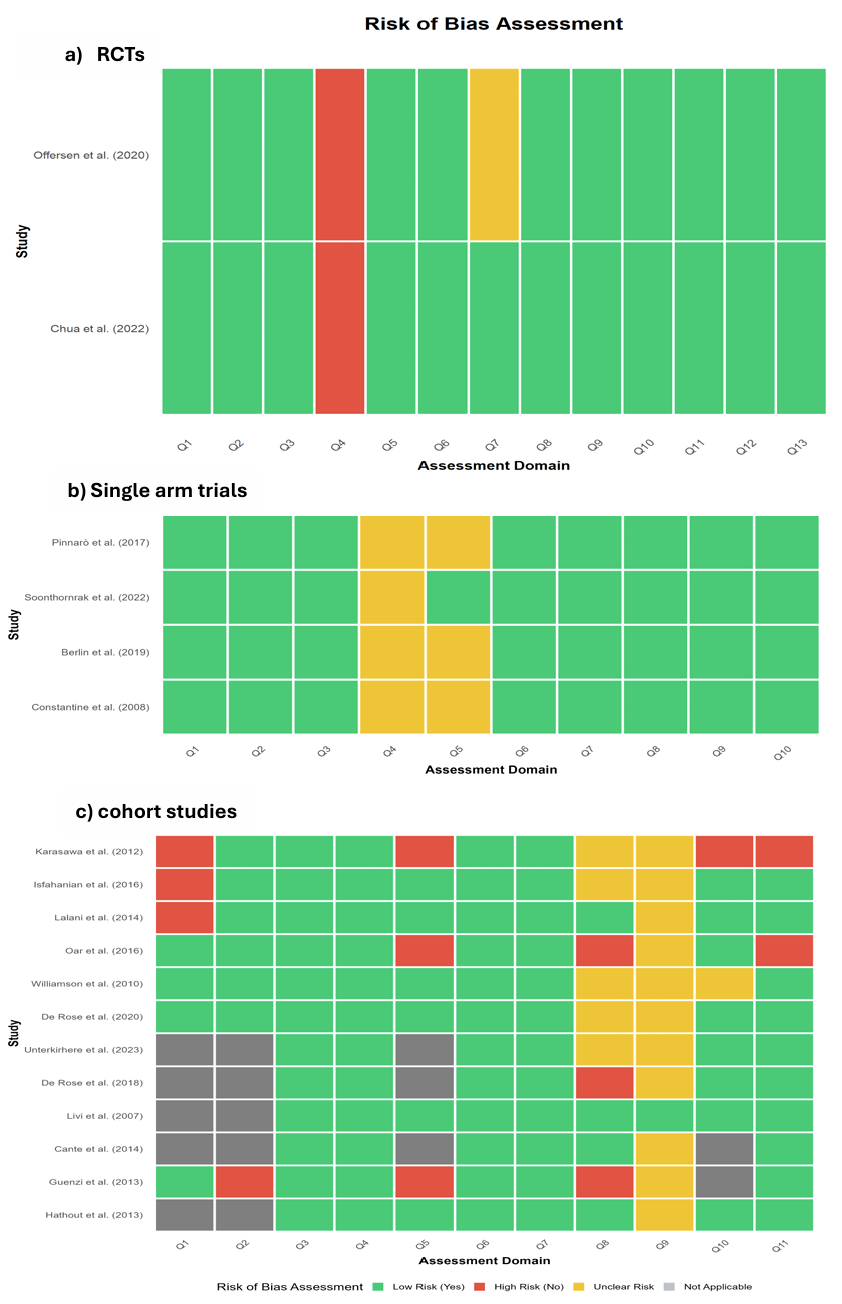

Supplement: Supplementary file 2 — Supporting Information 2 Figure S2. Risk of bias assessment of included studies. (a) Randomized controlled trials, (b) single‐arm trials, and (c) cohort studies. [file TBJ-2026-9456822-s006.png]
