# Supplementary material for: Effectiveness and Safety of Hypofractionated Radiotherapy in Patients With Ductal Carcinoma In Situ (DCIS)
Source: Breast J. 2026 Jun 8;2026:9456822. doi: 10.1155/tbj/9456822 (PMC13244251; doi:10.1155/tbj/9456822)
Supplement: Supplementary file 3 — Supporting Information 3 Table S1. Full search strategy for PubMed, Embase, WoS, and Scopus databases. [file TBJ-2026-9456822-s005.docx]

| **Table S1.** Full search strategy for PubMed, Embase, WoS, and Scopus databases. |
| --- |
| PubMed: 499 results, (July 12, 2025) |
| ("Carcinoma, Ductal, Breast"[Mesh] OR "ductal carcinoma in situ"[tiab] OR "DCIS"[tiab] OR "Intraductal carcinoma"[tiab] OR "non-invasive breast cancer"[tiab] OR "noninvasive breast cancer"[tiab] OR "pre-invasive breast cancer"[tiab] OR "preinvasive breast cancer"[tiab] OR "in situ breast carcinoma"[tiab] OR “in situ” [tiab]) AND  ("Radiotherapy"[Mesh] OR "Radiotherapy, Conformal"[Mesh] OR "radiotherapy"[tiab] OR "radiation therapy"[tiab] OR "RT"[tiab]) AND  (hypofr* [tiab] OR "hypofractionation"[tiab] OR "hypofractionated"[tiab] OR "short-course"[tiab] OR "abbreviated"[tiab] OR "accelerated"[tiab]) |
| Embase (Elsevier): 1119 results, (July 12, 2025) |
| ('Carcinoma, Ductal, Breast'/exp OR 'ductal carcinoma in situ':ti,ab OR DCIS:ti,ab OR 'Intraductal carcinoma':ti,ab OR 'non-invasive breast cancer':ti,ab OR 'noninvasive breast cancer':ti,ab OR 'pre-invasive breast cancer':ti,ab OR 'preinvasive breast cancer':ti,ab OR 'in situ breast carcinoma':ti,ab OR 'in situ':ti,ab) AND (Radiotherapy/exp OR 'Radiotherapy, Conformal'/exp OR radiotherapy:ti,ab OR 'radiation therapy':ti,ab OR RT:ti,ab) AND (hypofr*:ti,ab OR hypofractionation:ti,ab OR hypofractionated:ti,ab OR short-course:ti,ab OR abbreviated:ti,ab OR accelerated:ti,ab) |
| WoS Advanced: 439 results, (July 12, 2025) |
| (ALL="Carcinoma, Ductal, Breast" OR (TI="ductal carcinoma in situ" OR AB="ductal carcinoma in situ") OR (TI=DCIS OR AB=DCIS) OR (TI="Intraductal carcinoma" OR AB="Intraductal carcinoma") OR (TI="non-invasive breast cancer" OR AB="non-invasive breast cancer") OR (TI="noninvasive breast cancer" OR AB="noninvasive breast cancer") OR (TI="pre-invasive breast cancer" OR AB="pre-invasive breast cancer") OR (TI="preinvasive breast cancer" OR AB="preinvasive breast cancer") OR (TI="in situ breast carcinoma" OR AB="in situ breast carcinoma") OR (TI="in situ" OR AB="in situ")) AND (ALL=Radiotherapy OR ALL="Radiotherapy, Conformal" OR (TI=radiotherapy OR AB=radiotherapy) OR (TI="radiation therapy" OR AB="radiation therapy") OR (TI=RT OR AB=RT)) AND ((TI=hypofr* OR AB=hypofr*) OR (TI=hypofractionation OR AB=hypofractionation) OR (TI=hypofractionated OR AB=hypofractionated) OR (TI=short-course OR AB=short-course) OR (TI=abbreviated OR AB=abbreviated) OR (TI=accelerated OR AB=accelerated)) |
