# Supplementary material for: Effectiveness and Safety of Hypofractionated Radiotherapy in Patients With Ductal Carcinoma In Situ (DCIS)
Source: Breast J. 2026 Jun 8;2026:9456822. doi: 10.1155/tbj/9456822 (PMC13244251; doi:10.1155/tbj/9456822)
Supplement: Supplementary file 4 — Supporting Information 4 Table S2. Subgroup analysis of toxicities and oncological outcomes by study design. [file TBJ-2026-9456822-s001.docx]

**Table S2.** Subgroup analysis of toxicities and oncological outcomes by study design.

| **Outcome** | **Prospective % (95% CI); (Events/N)** | **Retrospective% (95%CI); (Events/N)** | **Trial% (95% CI); (Events/N)** | **p (Prospective vs. Retrospective)** | **p (Trial vs. Prospective)** | **p (Trial vs. Retrospective)** |
| --- | --- | --- | --- | --- | --- | --- |
| Cosmetic (Excellent/Good) | 87% [79%; 93%] (90/103) | 91% [77%; 97%] (346/346) | 94% [91%; 96%] (343/343) | 0.587 | 0.138 | **0.005** |
| Grade ≥2 Dermatitis | 6% [2%; 17%] (820/820) | 15% [11%; 19%] (346/346) | 5% [3%; 10%] (196/196) | **0.0039** | 0.522 | **<0.001** |
| Grade ≥2 Telangiectasia | 2% [0%; 7%] (103/103) | 0% [0%; 2%] (346/346) | 4% [1%; 23%] (310/310) | 0.2015 | 0.528 | 0.013 |
| Any-Grade Hyperpigmentation | 2% [0%; 7%] (103/103) | 0% [0%; 5%] (72/72) | 25% [0%; 99%] (92/92) | 0.4822 | 0.329 | 0.544 |
| Grade ≥2 Induration | 4% [1%; 10%] (103/103) | 1% [0%; 5%] (346/346) | 1% [0%; 7%] (92/92) | 0.2699 | 0.978 | 0.187 |
| Grade ≥2 Pain | 0% [0%; 1%] (717/717) | 20% [8%; 43%] (346/346) | 1% [0%; 5%] (196/196) | **<0.0001** | 0.120 | **<0.0001** |
| Grade ≥2 Pneumonitis | 0% [0%; 1%] (820/820) | — | — | — | — | — |
| Grade ≥2 Edema (Acute) | 11% [5%; 18%] (103/103) | 7% [2%; 15%] (72/72) | 0% [0%; 6%] (59/59) | 0.1567 | 0.874 | 0.039 |
| Shrinkage | 0% [0%; 4%] (103/103) | 18% [10%; 29%] (72/72) | — | **0.0084** | — | — |
| Grade ≥2 Fatigue | — | — | 1% [0%; 4%] (163/163) | — | — | — |
| Local Recurrence (3-year) | 853/853; 0% (0-1) | 530/530; 4% (3-6) | 308/308; 3% (2-7) | **p-value (Overall) :0.0014** | | |
| Local Recurrence (5-year) | — | 1258/1258; 7% (5-9) | 355/355; 2% (0-17) | p-value (Overall):0.5501 | | |
| Overall Survival (3-year) | 33/33; 100% (89-100) | 208/208; 99% (96-100) | 145/145; 99% (95-100) | p-value (Overall):0.9065 | | |
| Regional Nodal Recurrence (3-year) | 750/750; 1% (1-2) | 762/762; 2% (0-8) | 204/204; 1% (0-4) | p-value (Overall):0.4281 | | |
| Regional Nodal Recurrence (5-year) | — | 488/488; 0% (0-2) | 145/145; 1% (0-5) | p-value (Overall):0.6699 | | |
| Distant Metastasis (3-year) | 750/750; 1% (1-2) | 922/922; 1% (0-2) | 204/204; 1% (0-4) | p-value (Overall): 0.8780 | | |
| Distant Metastasis (5-year) | — | 794/794; 2% (0-5) | 145/145; 1% (0-5) | p-value (Overall):0.7869 | | |
| Breast Cancer-Specific Mortality (3-year) | 750/750; 1% (1-2) | 204/204; 1% (0-4) | — | p-value (Overall):0.6914 | | |
| Breast Cancer-Specific Mortality (5-year) | — | 56/56; 0% (0-6) | 145/145; 1% (0-5) | p-value (Overall):0.8907 | | |

**Values in bold indicate a significant difference between study design groups (p < 0.05).**Events/N: number of events per total participants; % (95% CI): proportion with 95% confidence interval
