# Supplementary material for: Effectiveness and Safety of Hypofractionated Radiotherapy in Patients With Ductal Carcinoma In Situ (DCIS)
Source: Breast J. 2026 Jun 8;2026:9456822. doi: 10.1155/tbj/9456822 (PMC13244251; doi:10.1155/tbj/9456822)
Supplement: Supplementary file 5 — Supporting Information 5 Table S3. Subgroup analysis of toxicities and oncological outcomes by radiotherapy technique. [file TBJ-2026-9456822-s008.docx]

**Table S3.** Subgroup analysis of toxicities and oncological outcomes by radiotherapy technique.

| **Outcome** | **3D-CRT Events/N; % (95% CI)** | **VMAT Events/N; % (95% CI)** | **IMRT Events/N; % (95% CI)** | **p-value (3D-CRT vs VMAT)** | **p-value (3D-CRT vs IMRT)** | **p-value (VMAT vs IMRT)** |
| --- | --- | --- | --- | --- | --- | --- |
| Cosmetic (Excellent/Good) | 480/541; 87% (77-93) | 260/274; 95% (92-97) | 31/33; 94% (80-99) | 0.0877 | 0.4458 | 0.1239 |
| Grade ≥2 Dermatitis | 39/951; 5% (2-11) | 42/274; 15% (12-20) | 2/33; 6% (1-20) | **0.0139** | **0.0139** | **0.0139** |
| Grade ≥2 Telangiectasia | 20/485; 3% (1-9) | 0/274; 0% (0-3) | — | 0.0877 | — | — |
| Any-Grade Hyperpigmentation | 52/234; 9% (0-87) | — | 0/33; 0% (0-11) | — | 0.4458 | — |
| Grade ≥2 Induration | 6/234; 3% (1-7) | 0/274; 0% (0-3) | 0/33; 0% (0-11) | 0.1239 | 0.1239 | 0.1239 |
| Grade ≥2 Pain | 9/848; 2% (0-10) | 82/274; 30% (25-36) | 0/33; 0% (0-11) | **0.0002** | **0.0002** | **0.0002** |
| Grade ≥2 Pneumonitis | 2/820; 0% (0-1) | — | — | — | — | — |
| Grade ≥2 Edema (Acute) | 16/234; 8% (5-14) | — | — | — | — | — |
| Shrinkage | 13/175; 4% (0-63) | — | — | — | — | — |
| Local Recurrence (3-year) | 10/1072; 2% (1-5) | 18/482; 4% (2-6) | — | 0.1776 | — | — |
| Local Recurrence (5-year) | 27/517; 5% (4-8) | 12/208; 6% (3-10) | — | 0.8170 | — | — |
| Overall Survival (3-year) | 145/145; 100% (97-100) | 206/208; 99% (97-100) | — | 0.7873 | — | — |
| Overall Survival (5-year) | 145/145; 100% (97-100) | 206/208; 99% (97-100) | — | 0.7873 | — | — |
| Regional Nodal Recurrence (3-year) | 1/1409; 0% (0-1) | 18/274; 7% (4-10) | — | **<0.0001** | — | — |
| Regional Nodal Recurrence (5-year) | 0/633; 0% (0-2) | — | — | — | — | — |
| Distant Metastasis (3-year) | 8/1361; 1% (0-2) | 4/482; 1% (1-3) | — | 0.4476 | — | — |
| Distant Metastasis (5-year) | 0/585; 0% (0-2) | 6/208; 3% (1-6) | — | **0.0169** | — | — |
| Breast Cancer-Specific Mortality (3-year) | 3/921; 1% (0-1) | — | — | — | — | — |
| Breast Cancer-Specific Mortality (5-year) | 0/201; 1% (0-4) | — | — | — | — |  |

**Values in bold indicate a significant difference between radiotherapy technique groups (p < 0.05).**Events/N: number of events per total participants; % (95% CI): proportion with 95% confidence interval
