# Supplementary material for: Effectiveness and Safety of Hypofractionated Radiotherapy in Patients With Ductal Carcinoma In Situ (DCIS)
Source: Breast J. 2026 Jun 8;2026:9456822. doi: 10.1155/tbj/9456822 (PMC13244251; doi:10.1155/tbj/9456822)
Supplement: Supplementary file 6 — Supporting Information 6 Table S4. Subgroup analysis of toxicities and oncological outcomes by treatment week. [file TBJ-2026-9456822-s002.docx]

**Table S4.** Subgroup analysis of toxicities and oncological outcomes by treatment week.

| **Outcome** | **Week 3 Events/N; % (95% CI)** | **Week 4 Events/N; % (95% CI)** | **Week 5 Events/N; % (95% CI)** | **p-value (Week 3 vs Week 4)** | **p-value (Week 3 vs Week 5)** |
| --- | --- | --- | --- | --- | --- |
| Cosmetic (Excellent/Good) | 655/745; 91% (84-95) | 90/103; 87% (79-93) | — | 0.3977 | — |
| Grade ≥2 Dermatitis | 76/1259; 7% (4-12) | 11/103; 11% (5-18) | — | 0.2838 | — |
| Grade ≥2 Telangiectasia | 18/656; 1% (0-6) | 2/103; 2% (0-7) | — | 0.7041 | — |
| Any-Grade Hyperpigmentation | 50/164; 9% (0-88) | 2/103; 2% (0-7) | — | 0.4831 | — |
| Grade ≥2 Induration | 2/438; 1% (0-3) | 4/103; 4% (1-10) | — | 0.1184 | — |
| Grade ≥2 Pain | 91/1259; 4% (1-17) | — | — | — | — |
| Grade ≥2 Pneumonitis | 2/717; 0% (0-1) | 0/103; 0% (0-4) | — | 0.7302 | — |
| Grade ≥2 Edema (Acute) | 5/131; 4% (1-22) | 11/103; 11% (5-18) | — | 0.2777 | — |
| Shrinkage | 13/72; 18% (10-29) | 0/103; 0% (0-4) | — | **0.0084** | — |
| Grade ≥2 Fatigue | 0/163; 1% (0-4) | — | — | — | — |
| Local Recurrence (3-year) | 23/1588; 3% (2-5) | 0/103; 0% (0-4) | — | 0.2245 | — |
| Local Recurrence (5-year) | 87/1613; 6% (5-7) | — | — | — | — |
| Overall Survival (3-year) | 384/386; 99% (98-100) | — | — | — | — |
| Regional Nodal Recurrence (3-year) | 19/1276; 2% (1-5) | — | 0/440; 0% (0-2) | — | 0.1098 |
| Regional Nodal Recurrence (5-year) | 0/193; 1% (0-4) | — | 0/440; 0% (0-2) | — | 0.3739 |
| Distant Metastasis (3-year) | 12/1436; 1% (1-2) | — | 0/440; 0% (0-2) | — | 0.1366 |
| Distant Metastasis (5-year) | 8/499; 2% (1-4) | — | 0/440; 0% (0-2) | — | **0.0432** |
| Breast Cancer-Specific Mortality (3-year) | 3/954; 1% (0-1) | — | — | — | — |
| Breast Cancer-Specific Mortality (5-year) | 0/201; 1% (0-4) | — | — | — | — |

**Values in bold indicate a significant difference between radiotherapy technique groups (p < 0.05).**Events/N: number of events per total participants; % (95% CI): proportion with 95% confidence interval
