# Supplementary material for: Effectiveness and Safety of Hypofractionated Radiotherapy in Patients With Ductal Carcinoma In Situ (DCIS)
Source: Breast J. 2026 Jun 8;2026:9456822. doi: 10.1155/tbj/9456822 (PMC13244251; doi:10.1155/tbj/9456822)
Supplement: Supplementary file 7 — Supporting Information 7 Table S5. Subgroup analysis of toxicities and oncological outcomes by fractionation schedule. [file TBJ-2026-9456822-s003.docx]

**Table S5.** Subgroup analysis of toxicities and oncological outcomes by fractionation schedule.

| **Outcome** | **13 Fractions Events/N; % (95% CI)** | **15 Fractions Events/N; % (95% CI)** | **16** **Fractions Events/N; % (95% CI)** | **20 Fractions Events/N; % (95% CI)** | **p-value (Overall)** |
| --- | --- | --- | --- | --- | --- |
| Cosmetic (Excellent/Good) | 56/72; 78% (66-87) | 316/333; 95% (92-97) | 269/264; 86% (55-97) | 90/103; 87% (79-93) | **<0.0001** |
| Grade ≥2 Dermatitis | 0/72; 0% (0-5) | 50/437; 10% (5-18) | 26/750; 4% (2-5) | 11/103; 11% (5-18) | **0.0010** |
| Grade ≥2 Telangiectasia | 0/72; 0% (0-5) | 0/333; 0% (0-2) | 20/354; 2% (0-7) | 2/103; 2% (0-7) | **0.0019** |
| Any-Grade Hyperpigmentation | 0/72; 0% (0-5) | 50/59; 85% (73-93) | 0/33; 0% (0-11) | 2/103; 2% (0-7) | **<0.0001** |
| Grade ≥2 Induration | 2/72; 3% (0-10) | 0/333; 0% (0-2) | 0/33; 0% (0-11) | 4/103; 4% (1-10) | 0.1672 |
| Grade ≥2 Pain | 5/72; 7% (2-15) | 83/437; 8% (1-42) | 3/750; 1% (0-1) | — | **0.0004** |
| Grade ≥2 Pneumonitis | — | — | 2/717; 0% (0-1) | 0/103; 0% (0-4) | 0.7302 |
| Grade ≥2 Edema (Acute) | 5/72; 7% (2-15) | 0/59; 0% (0-6) | — | 11/103; 11% (5-18) | 0.1567 |
| Shrinkage | 13/72; 18% (10-29) | — | — | 0/103; 0% (0-4) | **0.0084** |
| Grade ≥2 Fatigue | — | 0/163; 1% (0-4) | — | — | 0.7790 |
| Local Recurrence (3-year) | — | 23/719; 1% (1-3) | 4/765; 1% (0-14) | 0/103; 0% (0-4) | 0.2521 |
| Local Recurrence (5-year) | — | 12/312; 5% (3-9) | 69/1050; 7% (5-8) | — | 0.4452 |
| Overall Survival (3-year) | — | 381/383; 99% (98-100) | — | — | 0.9938 |
| Regional Nodal Recurrence (3-year) | — | 18/511; 5% (3-8) | 1/1205; 0% (0-1) | — | **<0.0001** |
| Regional Nodal Recurrence (5-year) | — | 0/145; 1% (0-5) | 0/488; 0% (0-2) | — | 0.6699 |
| Distant Metastasis (3-year) | — | 4/719; 1% (1-3) | 8/1157; 1% (0-2) | — | 0.4637 |
| Distant Metastasis (5-year) | — | 6/353; 2% (1-5) | 2/586; 1% (0-3) | — | 0.1125 |
| Breast Cancer-Specific Mortality (3-year) | — | 0/237; 1% (0-3) | 3/717; 0% (0-1) | — | 0.4180 |
| Breast Cancer-Specific Mortality (5-year) | — | 0/145; 1% (0-5) | 0/56; 0% (0-6) | — | 0.8907 |

**Values in bold indicate a significant difference between radiotherapy technique groups (p < 0.05).**Events/N: number of events per total participants; % (95% CI): proportion with 95% confidence interval
