# Supplementary material for: Effectiveness and Safety of Hypofractionated Radiotherapy in Patients With Ductal Carcinoma In Situ (DCIS)
Source: Breast J. 2026 Jun 8;2026:9456822. doi: 10.1155/tbj/9456822 (PMC13244251; doi:10.1155/tbj/9456822)
Supplement: Supplementary file 8 — Supporting Information 8 Table S6. Meta‐regression analysis of treatment‐related toxicities and oncological outcomes in breast cancer radiotherapy: association with clinical and dosimetric variables. [file TBJ-2026-9456822-s004.docx]

**Table S6.** Meta-regression analysis of treatment-related toxicities and oncological outcomes in breast cancer radiotherapy: association with clinical and dosimetric variables.

| **Outcome** | **EQD2 score** | **Boost** | **Grade 3** | **Sample** | **Hormone therapy** | **BED score** | **Left** | **Gy per Fraction** | **Supine** |
| --- | --- | --- | --- | --- | --- | --- | --- | --- | --- |
| Dermatitis | 0.618 | 0.231 | 0.426 | 0.208 | **0.00451** | 0.621 | - | 0.319 | - |
| Hyperpigmentation | 0.692 | **<0.001** | 0.181 | 0.811 | 0.895 | 0.692 | - | 0.826 | **<0.001** |
| Pain | **0.0248** | 0.222 | 0.404 | 0.265 | **<0.001** | **0.0252** | 0.455 | 0.833 | - |
| Telangiectasia | 0.125 | **0.0415** | **0.0493** | **<0.001** | **0.00825** | 0.125 | 0.553 | 0.245 | 0.797 |
| Induration | 0.404 | **0.0314** | 0.362 | 0.52 | 0.986 | 0.405 | 0.629 | 0.512 | 0.406 |
| Acute Edema | 0.979 | 0.0591 | - | 0.251 | - | 0.979 | - | 0.645 | 0.083 |
| Cosmetic | 0.502 | 0.279 | 0.372 | 0.18 | 0.658 | 0.398 | 0.801 | 0.693 | 0.418 |
| 5-year local recurrence rate | 0.357 | 0.501 | 0.922 | 0.202 | 0.412 | 0.383 | - | 0.271 | - |
| 3-year local recurrence rate | 0.204 | 0.192 | 0.297 | **<0.001** | 0.0662 | 0.205 | **0.00438** | 0.201 | - |
| 5-year overall survival | 0.365 | 0.221 | 0.29 | 0.877 | 0.211 | 0.365 | - | 0.797 | 0.815 |
| 3-year overall survival | 0.863 | 0.988 | 0.787 | 0.847 | - | 0.862 | - | 0.862 | 0.714 |
| 5-year breast cancer-specific mortality | 0.819 | 0.85 | - | 0.817 | - | 0.819 | - | 0.985 | - |
| 3-year breast cancer-specific mortality | 0.455 | 0.784 | - | 0.408 | - | 0.455 | - | 0.791 | - |
| 5-year distant metastasis rate | 0.114 | 0.147 | 0.356 | 0.0981 | 0.356 | 0.115 | - | 0.632 | 0.212 |
| 3-year distant metastasis rate | 0.704 | 0.679 | 0.553 | 0.968 | 0.701 | 0.705 | 0.823 | 0.788 | - |
| 5-year regional nodal recurrence rate | 0.784 | 0.845 | - | 0.311 | - | 0.782 | - | 0.406 | - |
| 3-year regional nodal recurrence rate | **<0.001** | 0.36 | 0.337 | 0.112 | 0.14 | **<0.001** | 0.222 | 0.882 | - |

**All values represent p-values from meta-regression analyses. Bold values indicate statistically significant associations (p < 0.05).**
**Abbreviations:** BED = Biological Effective Dose; EQD2 = Equivalent Dose in 2 Gy fractions.
Sample size: Total number of patients; Boost: Percentage receiving boost irradiation; Left: Percentage with left-sided tumors; Year: Publication year.
